# Supplementary material for: The interactome of intact mitochondria by cross-linking mass spectrometry provides evidence for coexisting respiratory supercomplexes
Source: Mol Cell Proteomics. 2017 Dec 8;17(2):216–32. doi: 10.1074/mcp.RA117.000470 (PMC5795388; doi:10.1074/mcp.RA117.000470)
Supplement: Supplemental Data [file supp_RA117.000470_133922_0_supp_23978_fzffwf.zip › spectra_annotation/mito_DR_spectra_annotation/182-1-7-1-7-1.pdf]

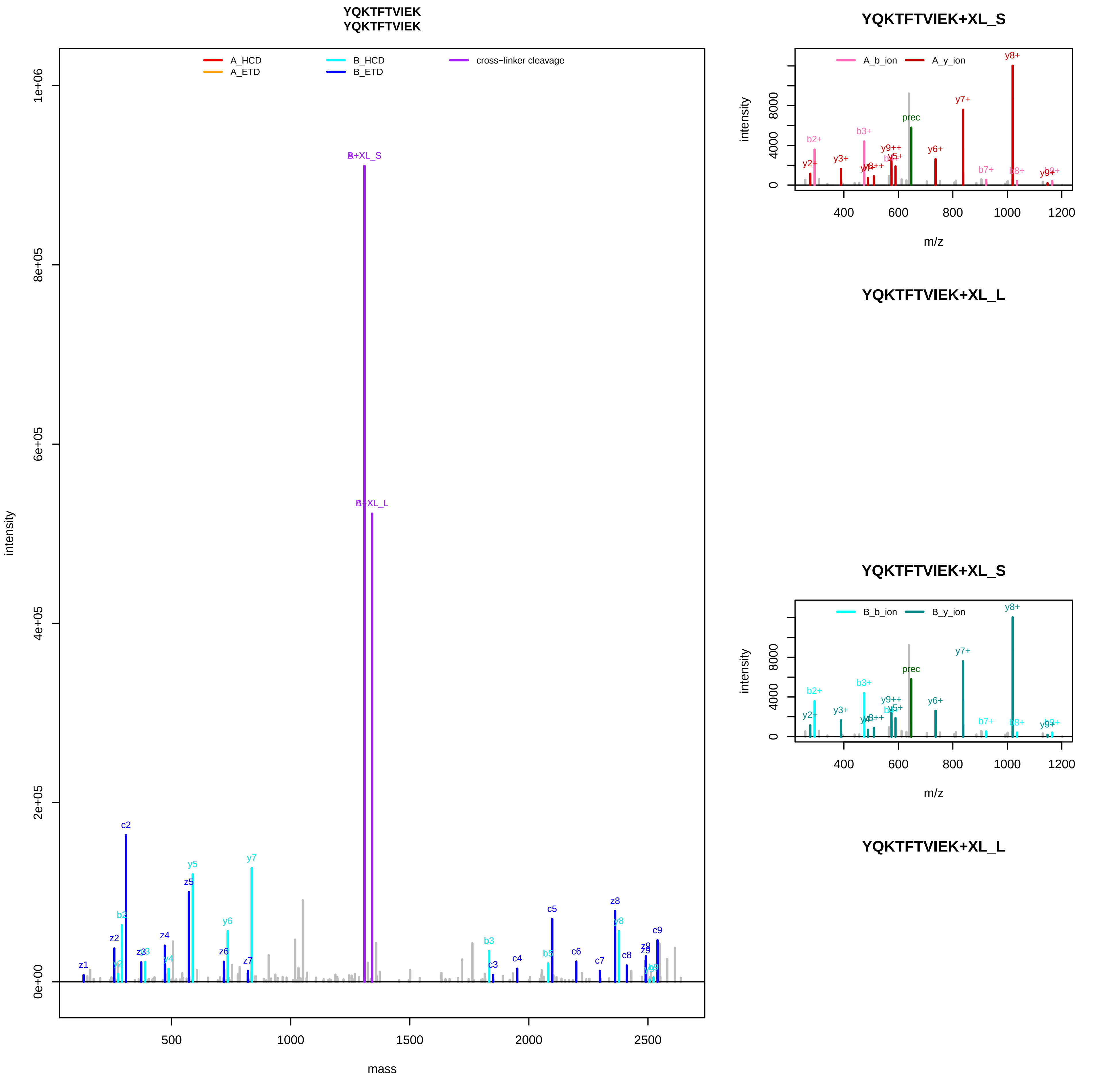

YQKTFTVIEK
YQKTFTVIEK
A_HCD
A_ETD
B_HCD
B_ETD
cross−linker cleavage
B+XL_S
B+XL_L
intensity
mass
0e+00
2e+05
4e+05
6e+05
8e+05
1e+06
500
1000
1500
2000
2500
z1
z2
c2
b2
z3
z4
z5
y5
z6
y6
z7
y7
y4
b3
c3
c4
b5
c5
c6
c7
z8
y8
c8
z9
c9
YQKTFTVIEK+XL_S
A_b_ion
A_y_ion
y2+
b2+
y3+
b3+
y9++
y5+
prec
y6+
y7+
y8+
b7+
b8+
b9+
y9+
m/z
0
4000
8000
400
600
800
1000
1200
YQKTFTVIEK+XL_L
YQKTFTVIEK+XL_S
B_b_ion
B_y_ion
YQKTFTVIEK+XL_L
